# Individual differences in the activity of executive function brain regions during number comparison

**DOI:** 10.1016/j.bbr.2025.115740

**Published:** 2025-07-22

**Authors:** Amanda Martinez-Lincoln, Daniel R. Leopold, Boman R. Groff, Darren J. Yeo, Erik G. Willcutt, Laurie E. Cutting, Marie T. Banich, Gavin R. Price

**Affiliations:** a Vanderbilt University, Peabody College of Human Development, United States; b University of Colorado Boulder, Institute of Cognitive Science, United States; c University of Colorado Boulder, Department of Psychology and Neuroscience, United States; d Nanyang Technological University, Division of Psychology, Singapore; e University of Exeter, Department of Psychology, United Kingdom

**Keywords:** Math, Symbolic, Nonsymbolic, Number comparison, Executive functions

## Abstract

Math skills require the integration of math-specific and domain-general skills, such as executive functions (EF). Neuroimaging studies consistently report intraparietal sulci activation during arithmetic tasks; however, activation of frontal brain regions associated with EF varies across studies. The discrepancies in brain regions associated with EF during math tasks may be due, in part, to variations amongst individuals and task demands. The current study examined neural activations associated with ratio effect in canonical math and EF regions in adolescents and subsequently examined how this activity was related to concurrently acquired behavioral measures of math ability and EF. Findings revealed differential relations between behavioral measures and neural ratio effects for symbolic (i.e., digits) vs. nonsymbolic (i.e., dot arrays) stimuli. The neural ratio effect during symbolic number comparison in the left inferior parietal lobe (IPL) and left inferior frontal junction (IFJ) correlated positively with an individual’s calculation scores. Similarly, the neural ratio effect during nonsymbolic comparison in the right and left inferior parietal lobes correlated positively with an individual’s math fluency. However, while a measure of an individual’s inhibitory control positively correlated with the nonsymbolic neural ratio effect in the left IFJ, working memory positively correlated with the symbolic neural ratio effect in the left IPL, left IFJ, left precentral gyrus, and left posterior dorsolateral prefrontal cortex. These findings suggest that the format of numerical information influences the neural systems engaged and that engagement varies with individual differences in math abilities.

## 1. Introduction

Children’s math skills are critical to their future educational outcomes, career readiness, and overall health and well-being [29,35,39, 57], yet many children struggle with math [47]. One of the factors that makes math skills difficult to acquire is their compositional nature, in that the acquisition and maintenance of mathematical skills require the integration of math-specific and domain-general skills, such as numerical magnitude processing and executive functions (EF) respectively [18, 28,68]. Individuals with poor mathematical skills may struggle with math-specific skills (e.g., numerical magnitude processing), domain-general skills (e.g., executive function), or both [28]. More recently, research has begun to ask whether, in addition to specific skill impairments, the *interaction* between domain-general and domain-specific skills may be a source of individual differences in math learning (e.g., [73]. To this end, a growing body of behavioral research suggests that the interplay between domain-specific math skills and executive functions (EF) are of particular importance for math skills development [51-53,65,73]. However, the underlying neural relationships across number formats are unclear. It is possible EF regions function differentially during magnitude processing in different number formats, and these relationships may relate differently to behavior [44]. It is difficult to distill such mechanisms from behavioral data alone because reaction time and accuracy are summary metrics that encapsulate the action of multiple cognitive processes. Neuroimaging, although subject to many limitations of its own, can provide a different perspective that helps to elucidate the cognitive mechanisms involved in tasks and the relationship between them, but to date, only a handful of studies have examined the neural mechanisms underlying the relationship between numerical processing systems and EF (e.g., [6,45,60,68,74]).

A large body of studies within the neuroimaging literature consistently report the neural activation of the left and right intraparietal sulci (IPS) during basic numerical and more advanced mathematical tasks [34,36,64]. At the same time, EFs are typically associated with neural activation in a range of frontal lobe regions [49,59]. However, frontal EF regions are typically not the focus of studies of numerical cognition, and as a result, little is known about how the neural mechanisms of EF function during numerical processing and how they relate to individual differences in math ability [44]. As previously mentioned, only a handful of neuroimaging studies have investigated the underlying neural relationship between EF and numerical processing [13,14,74]. In one of the first neuroimaging studies to examine this question, using a whole-brain analysis approach, Wilkey & Price [74] showed that attention and number processing interacted to affect activation in the inferior frontal gyrus, and that activity in this area, not parietal number processing areas, correlated with math abilities. Subsequently, Castaldi et al. [14] showed that the cortical representation of numerosity in the parietal lobe was amplified when numerosity was task-relevant, and Cai et al. [13] showed that numerosity-selective neurons only respond to their preferred numerosity when attention is focused on that numerosity. However, to the best of our knowledge, to date, no studies have investigated how the activity of EF and number-sensitive brain regions during numerical processing is related to behavioral indices of EF and math skills. The current study investigates the underlying EF neural associations involved in numerical processing and the relationship between numerical processing systems and EF by examining brain activation during nonsymbolic and symbolic numerical magnitude processing in canonical number processing and EF brain regions and relating such activation to individuals’ differences in math skills and EF. We examine numerical magnitude processing in a sample of older children and adolescents whose math instruction is no longer focusing on basic number information, such as magnitude, but instead concentrating on more complex mathematical problem solving [48]. This developmental period allows us to naturally control for basic numerical knowledge, while still having variability in math ability and EF to examine these relationships with numerical magnitude comparison.

### 1.1. Effects of format type

Different formats of numerical information (i.e., symbolic, nonsymbolic) may vary in the numerical and EF demands they place on an individual [40]. Across neuroimaging studies, typically developing individuals display activation in the right and left intraparietal sulci (IPS) during different types of number and math tasks including magnitude comparison and calculation [15,64]. However, the hemisphere of the IPS activation often varies and this may be due to the format of the number, with nonsymbolic processing typically associated with right hemisphere activation while symbolic processing relies more commonly on the left IPS [4]. However, little is known regarding the extent to which EF regions function differentially during symbolic vs nonsymbolic magnitude processing, and how those differences relate to behavior [44]. Accordingly, in this study we examine differences between neural activation during nonsymbolic and symbolic numerical processing in canonical EF and number processing brain regions.

### 1.2. Individual differences in math skills

One method to assess math skills is a magnitude comparison task, in which individuals are presented with two numerical representations and are asked to determine which numerical representation is greater. Centered on the Approximate Number System (ANS) which insinuates that numbers closer together in magnitude have overlapping neural representations [24], individuals tend to perform slower and less accurately when discriminating numerical representations closer together in magnitude than numerical representations that are further apart (for review see [61]. Consequently, the neural ratio between the numerical pairs can be modeled as a *ratio effect*, with a smaller ratio effect associated with increased ANS precision (e.g., [20,54]).

Individual differences in the function of numerical processing brain regions have been found to be associated with individual differences in math skills in a number of studies (e.g., [10,32,55]). However, the ways in which individual differences in the function of EF regions during numerical processing are associated with behavioral outcomes are less clear. Behavioral research suggests, for example, that math ability is associated with EF, such that individuals with low math ability tend to exhibit poor EF [11,22,40,50,51,60,73]. In addition, parallel to those with reading difficulties, individuals with math difficulties (MD) exhibit greater activation in executive functioning areas [19], suggesting increased effort. However, EF skills have been shown to act as a protective factor for students at risk for MD [56], once again highlighting the complex interplay between math and EF ability.

The relationship between math and EF may also differ depending on the format used to assess the processing of numerical magnitude. For instance, it has been shown in adults that nonsymbolic processing requires specific demands related to multistep strategies which in turn necessitate working memory and cognitive control skills [46]. Therefore, we expect brain activation in EF regions to be stronger during nonsymbolic than symbolic numerical processing, but for both formats, activation in EF and numerical brain regions will correlate positively with math skills.

### 1.3. Individual differences in EF

In the current study, we examine the association between individual differences in EF to brain activation in response to nonsymbolic and symbolic numerical processing in each of numerical processing and canonical EF brain regions. Previous research suggests that working memory and inhibitory control components of EF may be particularly relevant for different aspects of numerical and mathematical processing (e.g., [11,18,66,70]).

The Triple Code Model of numerical cognition suggests three number representations, including the Arabic numeral, auditory verbal word, and the nonsymbolic magnitude representation [23]. To determine which number magnitude is greater in a nonsymbolic and symbolic numerical processing, participants may transform Arabic numerals (i.e., symbolic number representations) into nonsymbolic magnitude representations, taxing the working memory system, which has a restricted capacity [7]. Thus, it is not surprising that working memory skills predict symbolic magnitude skills in young children [37]; yet, it remains unclear if this same pattern will be present in older children.

In contrast to working memory, much less is known about the relationship between math and inhibition. Similar to working memory, poor math ability is also associated with low inhibition skills [11]. Inhibition seems to play a particular role in suppressing incorrect solutions of inefficient strategies in solving calculation problems [9]. In nonsymbolic magnitude comparison tasks where the size of the dots is incongruent with the number of dots, inhibition can play a role in suppressing the distracting information (e.g., [16,27,31]); however, inhibition’s role in symbolic and controlled nonsymbolic magnitude comparison tasks remains unclear. Therefore, we investigated working memory and inhibitory control as distinct constructs in this study. We expect that individuals with higher scores in working memory and inhibitory control will show stronger activation of EF regions during both symbolic and nonsymbolic numerical comparison tasks.

### 1.4. Summary

In the current study, we examine the underlying EF neural relationships involved in numerical processing by assessing the association between individual differences in EF to brain activation in response to nonsymbolic and symbolic numerical processing in each of the numerical processing and canonical EF regions. We compare said activation between numerical formats, and examine the ways in which such activation is associated with individual differences in behavioral measures of math skills and EF. A richer characterization of the function of EF regions during numerical processing will contribute to our understanding of how the neural systems that underlie numerical magnitude processing interface with domain-general systems to produce individual differences in behavior.

## 2. Materials and methods

### 2.1. Participants

Participants for the study were recruited from a previously described longitudinal twin sample [68] at the University of Colorado Boulder. Participants were 151 children and adolescents (Mean age = 166.23 months; *SD* = 29.70, age range 120–203 months, 53.6 % female). The sample included 35 monozygotic (MZ; i.e., identical) and 101 dizygotic (DZ; i.e., fraternal) twin pairs, as well as 14 twins whose co-twin was either ineligible for scanning, removed during quality control, or declined to participate. Twin status was not the focus of the current study, nor was the sample large enough to perform a well-powered twin analysis. As a result, family status (i.e., twin pairs) was statistically controlled for in analyses. All participants reported normal or corrected-to-normal vision and had no history of neurological disorders. The racial and ethnic composition of the sample is 80.13 % White (11.26 % Hispanic), 0.66 % Other race, 17.22 % reporting more than one race (3.31 % American Indian or Alaska Native, 3.31 % Asian, 1.32 % Native Hawaiian or Pacific Islander, 5.96 % African American, and 3.97 % Other) and 2.65 % not reporting. Informed consent or assent was obtained from each individual prior to participation and all study procedures were approved by the Institutional Review Board at the University of Colorado Boulder.

### 2.2. Procedures

Participants completed a 2.5-hour session that consisted of approximately 50 min of behavioral testing and 90 min of neuroimaging. Parents of participants also completed questionnaires about their child’s behavioral and socioemotional functioning. fMRI was used to assess brain activity patterns while participants completed numerical comparison, lexical decision, and working memory tasks. The current study did not include the analyses of the lexical decision and the working memory task. The results of the numerical comparison tasks that assessed participants’ processing of symbolic and nonsymbolic qualities are the sole focus of the current study. Participants were paid for their time and received images of their anatomical brain scans for participating.

### 2.3. Behavioral assessments

Mathematical ability and executive functioning were assessed in participants using the following measures. For math, the Calculation and Math Facts Fluency subtests from the Woodcock-Johnson III (WJ-III; [76]) were administered to estimate participants’ ability to perform basic mathematical operations (untimed) and apply calculation skills to single-digit numbers (timed). The z-scores for Calculation and Math Facts Fluency were used for the analyses so that all relevant measures were on the same scale. Two tasks were used to measure executive functioning: the Delis-Kaplan Executive Function Systems (D-KEFS) Color-Word Interference Test [26] was used to assess inhibition and the 2-back task on an N-back, modeled on that used in the Human Connectome Project [8], was used to assess working memory. For the D-KEFS inhibition measure, the standard scores from the inhibition minus the color naming trials were calculated. The 2-back consisted of 8 blocks where participants indicated by button press whether the current stimulus matched the item presented 2 trials prior. Each 2.5 s trial consisted of a 250 ms fixation cross, 2000 ms stimulus presentation, and 250 ms inter-trial interval. There were three types of stimuli used: letters (A, C, E, H, J, M, P, T, X, Z), Arabic numerals (0–9), and nonsense shapes adapted from Vanderplas and Garvin [67]. See Table 1 for participants’ behavioral performance on measures.

### 2.4. MRI acquisition parameters

All imaging data was collected on a 3-Tesla Siemens Prisma MRI scanner with 32-channel parallel imaging using multi-band sequences at the University of Colorado Boulder. To reduce motion, foam padding was placed around the participant’s head within the head coil and a weighted blanket was placed over the participant’s legs. The order of acquisitions was distortion correction scans (time = 44 s), two resting state scans (time = 6 m:07 s each), functional N-back scan (the behavioral results of which were used for the working memory measure), T1 MPRAGE sequence, the symbolic number comparison task, the nonsymbolic number comparison task, the circle comparison task (not of interest in current paper), four Diffusion Tensor Imagining (DTI) scans (3 m:16 s, 3 m:28 s, 3 m:8 s, 3 m:0 s), and a functional lexical decision scan (not of interest in current paper). The functional number comparison scans were collected with the following parameters: TR = 460 ms, TE = 27.2 ms, FOV = 248 mm; slices = 82; multiband acceleration factor = 8; voxel size = 3 × 3 × 3 mm; flip angle = 44°; time = 3 m:58 s each. The structural images were collected using a T1-weighted high-resolution 3D magnetization prepared rapid gradient echo (MPRAGE) sequence acquired in the sagittal plane with the following parameters: TR = 2400 ms, TE = 2.07 ms, FOV = 256 mm; slices = 320; iPAT GRAPPA acceleration factor = 2; voxel size = 0.8 × 0.8 × 0.8 mm; flip angle = 8°; time = 7 m:02 s).

### 2.5. fMRI task

Participants completed one run each of a (1) symbolic and (2) nonsymbolic number comparison task using an event-related fMRI design. For each trial, participants were asked to respond as quickly and as accurately as possible to indicate whether an Arabic digit (symbolic) or dot array (nonsymbolic) was greater or less than five by pressing a button with either their right index or left index finger, respectively. Notably, the stimuli do not require participants to suppress incongruent visual information such as surface area, density, or convex hull, such as other nonsymbolic tasks [30,71], which could account for a differential role of inhibition across the formats. For each task, 72 trials were presented within a run. Six stimulus numerosities —2, 3, 4, 6, 7, or 8—occurred with equal probability (i.e., 12 trials per numerosity) in an intermixed and pseudo-random order (i.e., no more than 2 and 4 consecutive trials were of the same numerosity and same response, respectively). These numerosities yielded ratios of 0.4, 0.6, 0.625, 0.714, 0.8, and 0.833 with respect to reference value of five. To minimize the use of non-numerical visual cues for nonsymbolic number comparison, we controlled for visual parameters of dot arrays across trials by generating the stimuli using the script by Dehaene et al. [25]. Specifically, half of the set of dot arrays was controlled for the total surface area across numerosities such that dot size decreased with numerosity, and half of the set was controlled for dot size such that the total surface area increased with numerosity. The font size of the Arabic digits for the symbolic number comparison trials was selected to match the mean luminance of the dot arrays. Each trial began with an initial fixation of 500 ms, stimulus presentation of 750 ms, and a mean inter-trial interval (ITI) of 1630 ms (ranging from 1230 to 2030 ms, randomized in equal probability within each run). Each run also began with 9.50 s of fixation and ended with 10.50 s of fixation to estimate baseline activity. The total duration of each run was 3.79 min.

Response times and accuracy rates were recorded during the fMRI tasks. A Repeated Measures 2 × 6 ANOVA, with Format (Symbolic, Nonsymbolic) and Ratio (0.4, 0.6, 0.63, 0.71, 0.80, 0.83) was conducted to examine participant performance (*p*) on the fMRI task for response time (ms) and percent accuracy, using Eq. (1), where RT is response time and ER is the error rate [43].


(1) p=RT(1+2(ER))

### 2.6. fMRI preprocessing

Image data pre-processing was implemented using the FSL package (analysis group, FMRIB, Oxford, UK, http://www.fmrib.ox.ac.uk/fsl/), using standard preprocessing procedures: for each functional image, brain extraction using Brain Extraction Tool (BET), motion correction, distortion correction, spatial smoothing, and resampling. Motion correction using a 12 degree of freedom model were performed. For distortion correction, fieldmap images were derived from B0 mapping image pairs for each functional series. To ensure intensity stabilization, the initial 10 volumes of each functional series were removed. Two stages were used for image registration to spatially transform all functional series to MNI152 standard space; first, using a 6 degree of freedom model between a single band reference image and high-resolution structural scan, then using a 12 degree of freedom model between the subject’s high-resolution structural scan and MNI152 high-resolution scan. Denoising was performed using AROMA, using nonaggressive noise removal. Quality assurance and inclusion were conducted on participants’ motion graphs, signal-to-noise ratios, and brain coverage parameters, using a 0–3 scale for motion and a 0–2 scale for signal-to-noise/coverage quality via visual and quantitative inspection of participant data.

### 2.7. fMRI analysis

#### 2.7.1. General approach

Permutation-based testing was implemented using PALM [75] to reduce reliance upon statistical assumptions, control for the non-independence of twin pairs, and address multiple comparison problems with whole-brain analyses. Twin pairs were statistically controlled for using sign-flipping and exchangeability blocks within PALM. Main effects were subjected to 10,000 permutations [2] and all significant clusters were identified using threshold-free cluster enhancement [62] with a family-wise error rate of *p* < .05, corrected across contrasts. Group-level main effects for the different format types (i.e., symbolic, nonsymbolic) were first compared. A whole-brain approach was utilized, controlling for age and sex.

#### 2.7.2. Effects of individual differences in math ability and executive functioning

To test the degree to which individual differences in math and EF are related to brain activation in math and EF regions, the neural ratio effect for each of the symbolic and nonsymbolic number comparison tasks were examined. The neural ratio effect was modeled using the FSL FEAT package (analysis group, FMRIB, Oxford, UK, http://www.fmrib.ox.ac.uk/fsl/) in which the 3rd column of an Explanatory Variables (EV) File was parametrically varied based upon the ratio of the stimulus and referent (i.e., 5) using the smaller numerator divided by the large denominator. The lower-level model isolated this effect using 4 EVs: 1) the parametrically varied ratio effect for each accurate trial, from stimulus presentation to response times (i.e., the effect of interest), 2) the postresponse time duration for accurate trials, 3) error trials, and 4) each trial’s pre-stimulus fixation blocks. The difference between the stimulus types was run as a fixed effects model of symbolic minus nonsymbolic. For *a priori* Regions of Interest (ROIs), the percent signal change was extracted using the *flsmeants* command within a spheroid, with a radius of 5 mm (81 voxels/sphere in 2 × 2 × 2 mm^3^ functional space). The percent signal change was then correlated with behavioral math and EF measures in these *a priori* ROIs. Follow-up analyses that controlled for sex, age, and family status (i.e., twin pairs) were conducted for significant correlations.

#### 2.7.3. ROI selection

Canonical ROIs of executive function- and math-specific brain regions were selected based on prior literature. Five ROIs were selected for executive function [68,69], including the left precentral gyrus (lPCG), the left inferior frontal junction (lIFJ), the left inferior frontal gyrus (lIFG), the left anterior dorsolateral prefrontal cortex (alDLPFC), and the left posterior dorsolateral prefrontal cortex (plDLPFC). For math-specific regions, nonsymbolic numerical magnitude, symbolic numerical magnitude, and general numerical magnitude inferior parietal lobule (IPL) regions in the left and right hemispheres were selected based on the meta-analysis by Sokolowski, Fias, Ononye, et al. [64]. See Table 2 for the coordinates of specific ROIs for executive function and math.

## 3. Results

### 3.1. Behavioral performance in MRI tasks

**Fig. 1.**
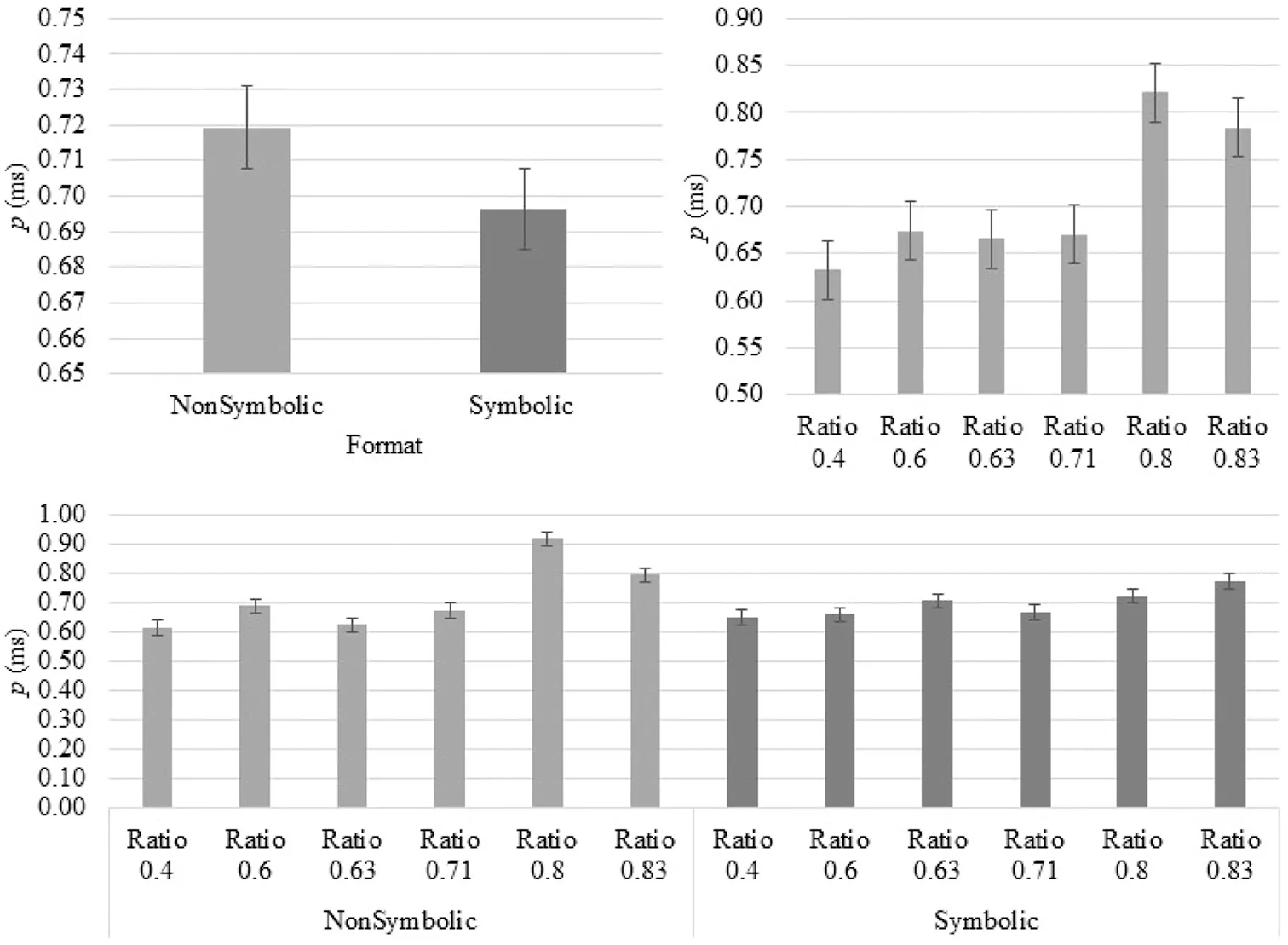
In-scanner behavioral performance (*p*).

Overall *p* (response time adjusted for accuracy) was greater for nonsymbolic than symbolic number comparisons (F(1172) = 8.352, *p* < 0.01) and for larger (numerically closer) than smaller (numerically further) ratios (F(5860) = 116.843, *p* < 0.01), see Fig. 1. There was also a greater ratio effect for nonsymbolic than symbolic number comparisons (F(5860) = 49.786, *p* < 0.01). See Table 1 for descriptive statistics of participants’ behavioral performance. This pattern of effects is in line with past reports in the literature.

### 3.2. Overall ratio effect

**Fig. 2.**
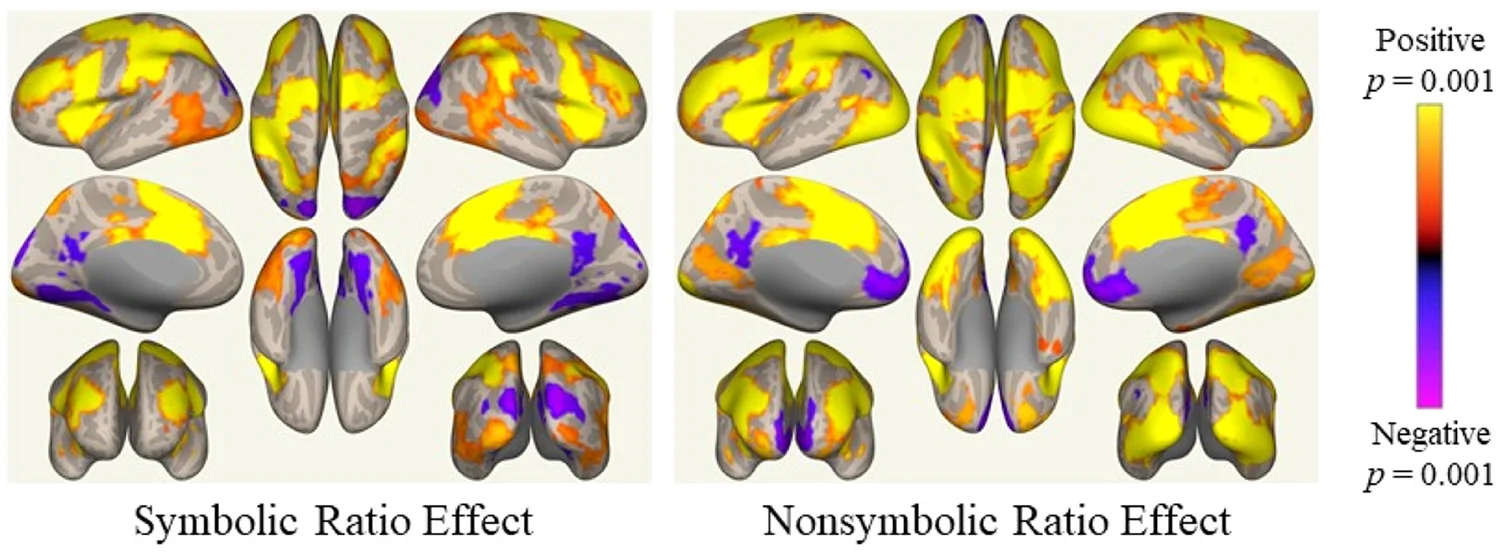
Ratio Effect.

As a preliminary analysis, we analyzed the comparisons of ratio effect *>* fixation for symbolic and nonsymbolic individually to verify that the number comparison task performed as expected for each format. Consistent with prior literature [17,21,36,44,64], the intraparietal sulcus was activated during both format types (Fig. 2; see also Table 3) in addition to activation in various pre-frontal regions [17,44,68]. Thus, the tasks each exhibited expected effects.

In the next sections, we discuss the relationship of individual differences in each of math and EF abilities with the neural ratio effects first for symbolic stimuli and then for non-symbolic stimuli.

### 3.3. Symbolic neural ratio effect

Findings revealed differential relations between individuals’ differences in math/EF ability and the neural ratio effect.

#### 3.3.1. Individual differences in math ability

**Fig. 3.**
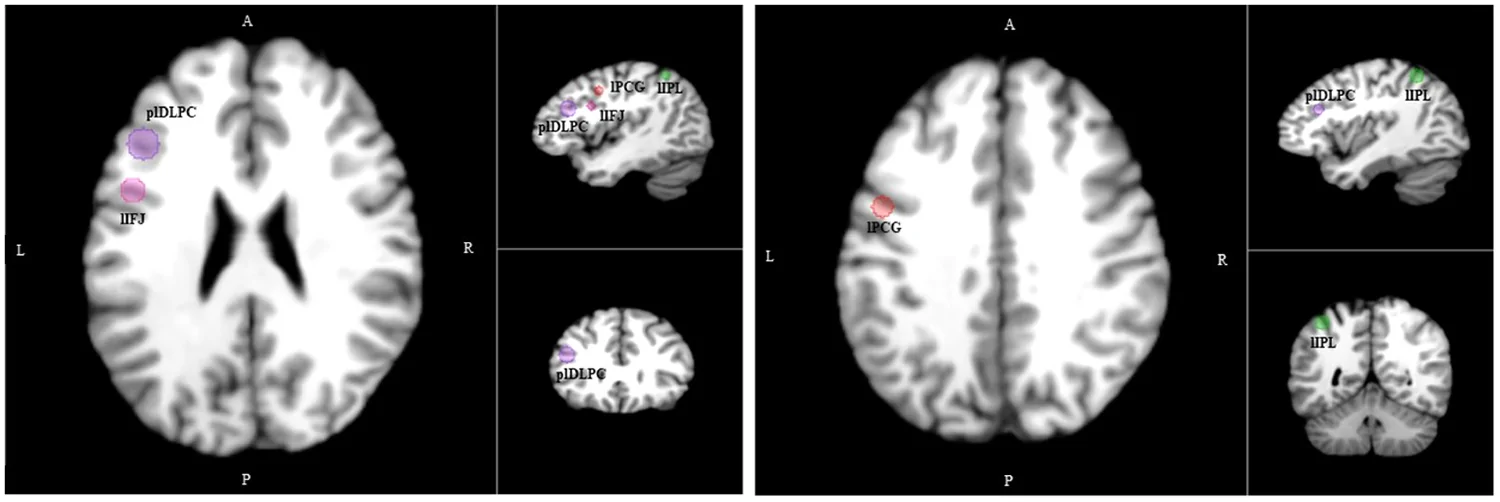
Two views of the significant ROIs for the correlations between symbolic ratio effect and math or EF ability, after controlling for sex, age, and family status. The left Precentral Gyrus (lPCG)is in red, left Inferior Frontal Junction (lIFJ) in pink, left Posterior Dorsolateral Prefrontal Cortex (plDLPFC) in purple, and the left Inferior Parietal Lobule (lIPL) is in green.

For the symbolic number comparison task, math ability was correlated with the ratio effect in brain regions associated with math and also in brain regions associated with EF (see Table 4 and Fig. 3). Specifically, calculation ability was positively correlated with activation associated with the ratio effect in the left IPL (*r* = 0.23, *p* < 0.01), even after controlling for sex, age, and family status (*r* = 0.22, *p* < 0.05). Calculation ability was also positively correlated with activation associated with the ratio effect in the left PCG (*r* = 0.18, *p* < 0.05), left IFJ (*r* = 0.27, *p* < 0.01), and left IFG ratio (*r* = 0.17, *p* < 0.05), but only the left IFJ remained significant after controlling for sex, age, and family status (*r* = 0.26, *p* < 0.01). Activation in the left IFJ was also positively correlated with math fluency (*r* = 0.25, *p* < 0.01), even after controlling for sex, age, and family status (*r* = 0.27, *p* < 0.01).

#### 3.3.2. Individual differences in EF ability

For the symbolic number comparison task, working memory scores were positively correlated with activation associated with the ratio effect in the left PCG (*r* = 0.28, *p* < 0.01), left IFJ (*r* = 0.29, *p* < 0.01), left posterior (*r* = 0.23, *p* < 0.05) and anterior DLPFC (*r* = 0.20, *p* < 0.05), and the left (*r* = 0.23, *p* < 0.05; *r* = 0.20, *p* < 0.05; *r* = 0.24, *p* < 0.05) and right IPL (*r* = 0.20, *p* < 0.05; *r* = 0.22, *p* < 0.05). Only the left PCG (*r* = 0.23, *p* < 0.01), left IFG (*r* = 0.21, *p* < 0.05), left posterior DLPFC (*r* = 0.17, *p* < 0.05) and left IPL (*r* = 0.22, *p* < 0.01) remained significant after controlling for sex, age, and family status. Inhibition scores did not correlate with the neural ratio effect in any region.

### 3.4. Nonsymbolic neural ratio effect

**Fig. 4.**
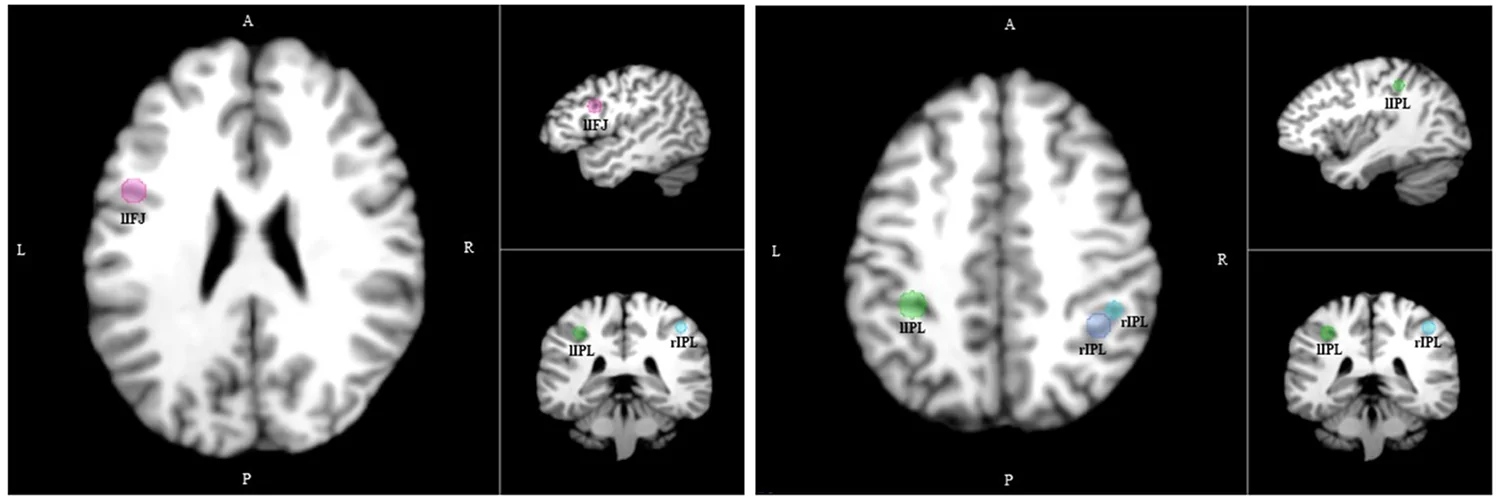
Significant ROIs for the correlations between nonsymbolic ratio effect and math or EF ability, after controlling for sex, age, and family status. The left Inferior Frontal Junction (lIFJ) in pink, the left Inferior Parietal Lobule (lIPL) is in green, and the right Inferior Parietal Lobule (rIPL) is in blue and light blue.

In contrast to the symbolic task, for the nonsymbolic number comparison task, math ability and EF ability each correlated with the activation associated with the ratio effect in brain regions associated with math, while EF ability only correlated with activation associated with the ratio effect in brain regions associated with EF (see Table 5 and Fig. 4).

#### 3.4.1. Individual differences in math ability

The activation associated with the ratio effects in two ROIs within the right IPL were positively correlated with math fluency (*r* = 0.22, *p* < 0.05; *r* = 0.22, *p* < 0.05; i.e., better math fluency was associated with more activation), even after controlling for sex, age, and family status (*r* = 0.19, *p* < 0.05; *r* = 0.21, *p* < 0.05). The left IPL was positively correlated with calculation scores (*r* = 0.19, *p* < 0.05) and math fluency (*r* = 0.23, *p* < 0.01), but only the math fluency (*r* = 0.22, *p* < 0.05) remained significant after controlling for sex, age, and family status.

#### 3.4.2. Individual differences in EF ability

For the nonsymbolic number comparison task, inhibition scores were positively correlated with activation associated with the ratio effect in the left IFG (*r* = 0.20, *p* < 0.05), even after controlling for sex, age, and family status (*r* = −0.18, *p* < 0.05). Working memory scores did not correlate with the neural ratio effect in any region.

## 4. Discussion

The current study adds to the literature exploring the underlying EF neural associations involved in numerical processing and the relationship between numerical processing systems and EF. Specifically, the findings showed that the numerical format in which mathematical information is presented influences the neural systems engaged and that the engagement of these regions varies with the level of mathematical ability as well as the level of EF. Overall, the neuroimaging results showed significant ratio effects in a range of parietal and frontal lobe brain regions, consistent with a large body of previous literature (for review see [5,63]). Our results also showed that individuals with better calculation abilities exhibited greater activation associated with the neural ratio effect during symbolic number comparison in the left inferior parietal lobe and left inferior frontal junction. Similarly, individuals with better math fluency exhibited greater activation associated with the neural ratio effect during nonsymbolic comparison in the right and left inferior parietal lobes. These findings are again consistent with a body of previous literature on children (e.g., [10,21,38,72] and young adults [72]). The current study provides evidence for a similar pattern of individual differences in math skills modulating the activation of brain regions associated with math in older children and adolescents.

The IPS plays a central role in the processing of numerical magnitude, however, variation in the hemisphere of the IPS activation has been frequently observed as a function of the format of the number within the task [4]. Our results support a body of previous work regarding hemispheric differences in numerical magnitude processing in relation to math skills, in that the neural correlates of symbolic processing correlated with math skills (e.g., see [77] in both hemispheres (albeit it is for the inferior temporal regions that are thought to be strongly connected with parietal regions, e.g., [1,3,33]), while those for symbolic magnitude processing only correlated in the left parietal lobe (e.g., [10,55,58]). This aligns with the perspective that symbolic and nonsymbolic are processed with distinct as well as overlapping number processing systems, rather than a single system [12,64]. Moreover, the current study provided evidence of this distinct number system in adolescents who completed the task in both numerical formats.

Most notably, our results indicated that the association between individual differences in EF differed across numerical formats. Behavioral studies suggest that children with higher work memory scores perform better and show greater growth in magnitude comparison tasks [41,53,9]. Our neural results align with these behavioral findings such that working memory scores correlated with the symbolic neural ratio effect in several EF brain regions, including the left precentral gyrus, inferior frontal junction, and dorsolateral prefrontal cortex. They also correlated with activity in the bilateral inferior parietal lobes. This finding provides further evidence supporting the emerging body of work suggesting that the capacity for neurocognitive EF systems to act upon numerical information may be important for various math skills [66,70].

Interestingly, working memory scores did not correlate with the nonsymbolic neural ratio effect. These findings offer new insights into the interplay between working memory and numerical magnitude processing across number formats. Specifically, they show that the neural task demands of working memory during magnitude comparisons differ across number formats, suggesting that working memory may be coupled more closely to symbolic than nonsymbolic numerical representations in older children and adolescents. Perhaps, this could indicate that older children and adolescents are implementing an additional step for symbolic number representations, such as mapping Arabic numerals (i.e., symbolic number representations) onto nonsymbolic magnitude representations. Additionally, this distinct working memory effect across numerical representations could support the Triple Code Model of numerical cognition, which designates three discrete number representations [23]. Although further research is needed, these neural findings began to unravel the intricacies of working memory and magnitude comparisons across different types of number formats.

Similar to working memory, behavioral studies also indicate a relationship between inhibition scores and magnitude comparison tasks in children [16,27,31]. Our results indicate that inhibition scores did not correlate with the symbolic neural ratio effect in any regions. They did, however, correlate positively with activation during the nonsymbolic ratio effect in the left inferior frontal gyrus. Again, these results offer new insights into the interplay between EF and numerical magnitude processing across number formats. Perhaps, providing additional support for the discrete number representations in the Triple Code Model of numerical cognition [23]. The relationship of inhibition for only the nonsymbolic ratio effect is, in some ways, unsurprising, given the sometimes conflicting visual processing demands present in nonsymbolic comparison trials that are not present in symbolic processing trials (for review see, [63]). However, importantly, in the current study, the paradigm (i.e., smaller or larger than 5) did not require participants to suppress incongruent visual information such as surface area, density, or convex hull [30,71] in order to select the numerically larger set. Therefore, the fact that the ratio effect of the nonsymbolic numerical comparison correlated with individuals’ inhibition skills speaks to a possible connection to learning to extract numerosity from visual arrays (i.e., in the environment) that requires the application of EF mechanisms that are not required in the learning of numerical symbols. Nonetheless, future studies will need to examine whether numerical symbols draw upon EF mechanisms during learning, but reliance on such systems gradually diminishes over development.

### 4.1. Limitations and future directions

Limitations should be considered when interpreting the findings of the current study. The current study measured EF using two tasks: one for inhibition and one for working memory. Future studies should examine whether individual differences in the current findings correspond when measuring EF using different methods. Our nonsymbolic magnitude task also included small number values that can be subitized [42]; therefore, our findings may not be generalized to other ANS tasks using larger number values. Future studies with larger twin samples may also examine the heritability of the effects in the current study to determine if individual differences are likely due to environment or genetics.

### 4.2. Conclusion and implications

In sum, the current study provides new insights into the complex interplay between EF and numerical magnitude processing across number formats, showing that the relationship between EF systems and number systems varies across number formats in terms of their relevance for math skills development. Most notably, our findings indicated that the association between individual differences in EF differed across numerical formats. Specifically, while individuals’ inhibitory control positively correlated with the nonsymbolic neural ratio effect in the left IFJ, working memory positively correlated with the symbolic neural ratio effect in the left IPL, left IFJ, left precentral gyrus, and left posterior dorsolateral prefrontal cortex. These findings suggest that the format of numerical information influences the neural systems engaged and that engagement varies with individual differences in math and EF abilities.

## Figures and Tables

**Table 1:** Descriptive Statistics.

	Mean (SD)	Range
**Age (months)**	166.23 (29.74)	120–203
**2-back Accuracy**	84.4 (12.5)	10.9–100
**D-KEFS Inhibition-Color SS**	10.00(2.16)	5–16
**WJ-III Calculation**	0.02(1.02)	−3.29–2.22
**WJ-III Math Fluency**	−0.01(0.99)	−2.42–2.16
**Nonsymbolic Overall *p* (ms)**	0.71 (0.17)	0.48–1.53
**Nonsymbolic Overall RTs (ms)**	0.59(0.01)	0.58–0.61
**Nonsymbolic Overall Accuracy**	0.90(0.01)	0.88–0.91
**Symbolic Overall *p* (ms)**	0.68 (0.17)	0.43–1.37
**Symbolic Overall RTs (ms)**	0.60(0.01)	0.58–0.61
**Symbolic Accuracy**	0.91(0.01)	0.90–0.93

*Note.* Delis-Kaplan Executive Function Systems (D-KEFS); SS = standard scores; WJ-III = Woodcock-Johnson III; *p* = performance; ms = milliseconds; RTs = response times; SD = standard deviation.

**Table 2:** ROIs.

Hemisphere	Brain Region	X	Y	Z
*Executive Function*				
Left	Precentral Gyrus (lPCG)	−46	2	38
Left	Inferior Frontal Junction (lIFJ)	−46	8	26
Left	Inferior Frontal Gyrus (lIFG)	−48	18	24
Left	Anterior Dorsolateral Prefrontal Cortex (alDLPFC)	−44	34	28
Left	Posterior Dorsolateral Prefrontal Cortex (plDLPFC)	−42	26	24
*Format-independent Numerical Magnitude Processing*				
Right	Inferior Parietal Lobule (rIPL)	38	−44	44
Left	Inferior Parietal Lobule (lIPL)	−34	−36	42
*Nonsymbolic-Specific Numerical Magnitude Processing*				
Right	Inferior Parietal Lobule (rIPL)	44	−38	46
Right	Inferior Parietal Lobule r(IPL)	38	−48	48
*Symbolic-Specific Numerical Magnitude Processing*				
Left	Inferior Parietal Lobule (lIPL)	−34	−52	36
Left	Inferior Parietal Lobule (lIPL)	−42	−44	38
Left	Inferior Parietal Lobule (lIPL)	−40	6	46
Left	Inferior Parietal Lobule (lIPL)	−38	−50	50
Right	Inferior Parietal Lobule (rIPL)	36	−44	42

*Note.* Each ROI was sphere-shaped with a radius of 5 mm (81 voxels/sphere in 2 × 2 X 2 mm^3^ functional space).

**Table 3:** Ratio Effect.

Cluster	Voxels	*p*	CoG x	CoG y	CoG z	Hemi- sphere	Anatomical Region
*Symbolic Ratio Effect*							
3	44145	.0001	−8.7	−3.6	28.1	L	cingulate gyrus
2	10713	.0001	42.6	−48.8	14.6	R	angular gyrus, middle temporal gyrus
1	38	.0479	−19.9	0.1	−14.2	L	cerebral cortex, amygdala
−2	4266	.0017	11.4	−79.1	15.8	R	intracalcarine cortex
−1	2291	.0069	−13	−56.4	3.16	L	lingual gyrus, precuneous cortex, cingulate cortex
*Nonsymbolic Ratio Effect*							
2	94087	.0001	1.7	−25.3	16.6	R	cingulate gyrus
1	79	.0447	−14.3	−40.3	68.1	L	postcentral gyrus
−3	2528	.0029	0	50.3	−2.4	L, R	paracingulate gyrus, frontal medial cortex
−2	855	.0079	−1.2	−52.3	24	L	cingulate gyrus, precuneous cortex
−1	24	.0447	−43.4	−66.2	30.8	L	lateral occipital cortex

*Note.* Positive and negative brain clusters correspond with activation reflected in Fig. 2. L = left; R = right

**Table 4:** Symbolic Ratio Effect.

ROI Region	ROI (X, Y, Z)	Calculation	Math Fluency	Working Memory	Inhibition
*Executive Function Regions*					
1PCG	−46, 2, 38	0.18*	0.06	0.28**	0.14
lIFJ	−46, 8, 26	0.27**	0.25**	0.29**	0.08
lIFG	−48, 18, 24	0.17*	0.16	0.15	0.13
plDLPFC	−42, 26, 24	0.13	0.14	0.23*	0.04
alDLPFC	−44, 34, 28	0.13	0.07	0.20*	0.02
*Format-independent Numerical Magnitude Processing Regions*					
Left IPL	−34, −36, 42	0.1	0.14	0.23*	0.04
Right IPL	38, −44, 44	0.12	0.07	0.20*	0.04
*Symbolic-Specific Numerical Magnitude Regions*					
Left IPL	−34, −52, 36	0.06	0.04	0.07	0.01
Left IPL	−42, −44, 38	0.01	−0.02	0.20*	0.06
Left IPL	−40, 6, 46	0.09	0.09	0.05	0.06
Left IPL	−38, −50, 50	0.23**	0.1	0.24*	0.04
Right IPL	36, −44, 42	0.12	0.06	0.22*	0.08

*Note*.

* Correlation significant at 0.05 level

** Correlation significant at 0.01 level.

**Bold** correlation remained significant at 0.05 level after controlling for sex, age, and family status.

**Table 5:** Nonsymbolic Ratio Effect.

ROI Region	ROI (X, Y, Z)	Calculation	Math Fluency	Working Memory	Inhibition
*Executive Function Regions*					
1PCG	−46, 2, 38	0.13	0.10	0.13	0.12
lIFJ	−46, 8, 26	0.11	0.07	0.13	**0.20** *
lIFG	−48, 18, 24	0.12	0.03	0.07	0.03
plDLPFC	−42, 26, 24	0	0.11	−0.04	0.07
alDLPFC	−44, 34, 28	−0.01	0.09	0.12	0.01
*Format-independent Numerical Magnitude Processing Regions*					
Left IPL	−34, −36, 42	0.19*	**0.23** **	0.13	0.11
Right IPL	38, −44, 44	−0.04	**0.22** *	0.12	0.11
*Nonsymbolic-Specific Numerical Magnitude Processing Regions*					
Right IPL	44, −38, 46	−0.08	**0.22** *	0.07	0.08
Right IPL	38, −48, 48	−0.09	0.17	0.13	0.14

*Note*.

* Correlation significant at 0.05 level

** Correlation significant at 0.01 level.

**Bold** correlation remained significant at 0.05 level after controlling for sex, age, and family status.

## Data Availability

Data will be made available on request.
